# 

**Published:** 1853-04

**Authors:** 


No. XI. APRIL. Vol. VIII.

### BUFFALO MEDICAL JOURNAL AND MONTHLY REVIEW OF MEDICAL AND SURGICAL SCIENCE.

EDITED BY AUSTIN FLINT, M. D.

BUFFALO:
PUBLISHED BY JEWETT, THOMAS & CO.
Commercial Advertiser Buildings.
1853

*Price* $2.50 *per annum* . . . . *in Advance.*

